# Enhanced cortical tracking of unfamiliar languages in both monolinguals and bilinguals

**DOI:** 10.64898/2026.09.21.753321

**Published:** 2026-09-28

**Authors:** Qianxun Zheng, Farhin Ahmed, Aaditya Chopra, Bonnie K. Lau

## Abstract

Humans routinely encounter speech in languages they have never heard, yet how the brain responds to such input and whether bilingual experience shapes this response remains unknown. Here, we used electroencephalography (EEG) and temporal response function (TRF) modeling to examine cortical tracking of the speech envelope in 24 English-monolingual and 24 English-Mandarin bilingual adults. Participants listened to naturally produced continuous speech in three languages: English (familiar to all), Mandarin (familiar to bilinguals only), and Vietnamese (unfamiliar to all). We report two main findings. First, both monolinguals and bilinguals showed enhanced cortical tracking for unfamiliar relative to familiar languages, evidenced by higher EEG prediction accuracy (PA). Monolinguals showed enhanced tracking for both Mandarin and Vietnamese, whereas bilinguals showed enhancement only for Vietnamese, consistent with Mandarin being a familiar language for this group. This finding suggests that enhanced cortical encoding of unfamiliar speech is a general property of the listening brain, not a signature of listening to a non-native language or reduced language proficiency. Second, bilinguals strikingly showed stronger cortical tracking than monolinguals overall, in both PA and TRF peak weights, with the TRF peak weight advantage present across all three languages, suggesting a difference in how bilingual experience shapes the neural encoding of speech. These findings have implications for understanding how the brain navigates the linguistic diversity of everyday life in an increasingly global, multilingual world.

**SIGNIFICANT STATEMENT:**

More than half the world speaks more than one language, and most people regularly hear speech they cannot understand. How the brain responds to such speech, and whether bilingualism matters, is unclear. We recorded brain activity from monolingual and bilingual adults listening to familiar and unfamiliar speech. Both groups showed enhanced tracking of unfamiliar speech compared to familiar speech and bilinguals showed stronger tracking than monolinguals overall, suggesting a potential difference in how bilingual brains process speech. These findings advance our understanding of how the brain navigates speech in a multilingual world.

## Full Text Availability

The license terms selected by the author(s) for this preprint version do not permit archiving in PMC. The full text is available from the preprint server.

